# The metabolic switch can be activated in a recombinant strain of *Streptomyces lividans* by a low oxygen transfer rate in shake flasks

**DOI:** 10.1186/s12934-018-1035-3

**Published:** 2018-11-28

**Authors:** Ramsés A. Gamboa-Suasnavart, Norma A. Valdez-Cruz, Gerardo Gaytan-Ortega, Greta I. Reynoso-Cereceda, Daniel Cabrera-Santos, Lorena López-Griego, Wolf Klöckner, Jochen Büchs, Mauricio A. Trujillo-Roldán

**Affiliations:** 1 Programa de Investigación de Producción de Biomoléculas, Unidad de Bioprocesos, Departamento de Biología Molecular y Biotecnología, Instituto de Investigaciones Biomédicas, Universidad Nacional Autónoma de México, AP. 70228, CP. 04510 Ciudad de México, Mexico; 2 Department of Biochemical Engineering (AVT.BioVT), RWTH Aachen University of Technology, Forckenbeckstraße 51, 52074 Aachen, Germany; 3 Bayer AG, Engineering and Technology, Chempark, 51368 Leverkusen, Germany

**Keywords:** Metabolic switch, Orbital shaking, *Streptomyces lividans*, Oxygen transfer rate, Recombinant glycoproteins, Shaken bioreactors, Undecylprodigiosin

## Abstract

**Background:**

In *Streptomyces*, understanding the switch from primary to secondary metabolism is important for maximizing the production of secondary metabolites such as antibiotics, as well as for optimizing recombinant glycoprotein production. Differences in *Streptomyces lividans* bacterial aggregation as well as recombinant glycoprotein production and *O*-mannosylation have been reported due to modifications in the shake flask design. We hypothetized that such differences are related to the metabolic switch that occurs under oxygen-limiting conditions in the cultures.

**Results:**

Shake flask design was found to affect undecylprodigiosin (RED, a marker of secondary metabolism) production; the RED yield was 12 and 385 times greater in conventional normal Erlenmeyer flasks (NF) than in baffled flasks (BF) and coiled flasks (CF), respectively. In addition, oxygen transfer rates (OTR) and carbon dioxide transfer rates were almost 15 times greater in cultures in CF and BF as compared with those in NF. Based on these data, we obtained respiration quotients (RQ) consistent with aerobic metabolism for CF and BF, but an RQ suggestive of anaerobic metabolism for NF.

**Conclusion:**

Although the metabolic switch is usually related to limitations in phosphate and nitrogen in *Streptomyces* sp., our results reveal that it can also be activated by low OTR, dramatically affecting recombinant glycoprotein production and *O*-mannosylation and increasing RED synthesis in the process.

**Electronic supplementary material:**

The online version of this article (10.1186/s12934-018-1035-3) contains supplementary material, which is available to authorized users.

## Background

The metabolic switch from the exponential to the stationary phase in the growth of *Streptomyces* sp. has been widely studied for the maximization of secondary metabolite production [1–8]. In addition, the primary products may also be of great value, such as recombinant glycoproteins produced by *Streptomyces lividans* [9–15].

The switch from primary to secondary metabolite biosynthesis normally causes a decrease in biomass, enhancing the carbon flux through the pentose phosphate pathway to increase NADPH generation [3, 16]. The secondary metabolites formed depend on the primary metabolites, such as glucose-6-phosphate, glyceraldehyde-3-phosphate, acetyl-CoA, α-ketoglutarate, and oxaloacetate, that serve as biosynthesis precursors and are generated during central carbon metabolism [17]. Furthermore, this metabolic switch has been associated with depletion of carbon, nitrogen, and phosphate sources in conventional shake flask cultures of *S. coelicolor* [2, 5, 18, 19]. During the metabolic switch, genes related to ribosomal proteins, protein biosynthesis, and nitrogen metabolism are downregulated, while those related to antibiotic biosynthesis (actinorhodin [ACT] and undecylprodigiosins [REDs]), as well as those related to the biosynthesis of several amino acids, are upregulated [3]. RED production has been proposed as a model for the prediction of secondary metabolism, as it is a red pigment that is easy to detect [20]. Moreover, bacterial RED and some of its synthetic derivatives have antitumor, antimicrobial, and antimalarial activities [21–27].

Previously, we reported the effect of the flask design on the production of a recombinant glycoprotein produced in *S. lividans* (APA, also known as alanine and proline-rich secreted protein or 45/47 kDa glycoprotein, an antigen from *Mycobacterium tuberculosis*) and its *O*-mannosylation [11]. Using the same operational conditions (150 rpm, shaking diameter of 2.5 cm, 250-mL flasks containing 50 mL of culture), ~ 0.81 mg/L of APA was produced in baffled (BF) and coiled flasks (CF, with a stainless-steel spring at the bottom of a normal flask), while ~ 0.51 mg/L of APA was obtained in normal standard Erlenmeyer flasks (NF) (Table 1). Moreover, up to five mannose residues were found attached to the C-terminal in cultures with smaller aggregates (BF and CF), while only two mannose residues were found in NF [11]. The microorganism morphology was also affected by the flask design (Table 1); smaller pellets were found in BF and CF (diameters of 0.23 ± 0.06 and 0.16 ± 0.05 mm, respectively) compared with those found in NF (1.57 ± 0.41 mm). These observations were attributed to differences in the oxygen transfer and volumetric power input (P/V) transferred to the culture medium [15]. Our group reported that more than double the power input was delivered to BF and CF (~ 0.51 W L^−1^ and ~ 0.44 W L^−1^) compared with NF (~ 0.20 W L^−1^) [15]. However, in shake flasks, momentum and mass transfer phenomena cannot be easily measured or controlled independently [28].

**Table 1:** Previously reported stoichiometric and kinetic data of recombinant glycoprotein production in *S. lividans* (APA, also known as alanine and proline-rich secreted protein or 45/47 kDa glycoprotein, an antigen from *Mycobacterium tuberculosis*) and its *O*-mannosylation cultured in shake flasks (nominal volume of 250 mL and 50 mL filling volume, incubated at 30 °C at a shaking frequency of 150 rpm)

Parameter	Shake flask			References
	Normal	Coiled	Baffled	
Pellet area (mm^2^)	2.11 ± 1.22	0.04 ± 0.02	0.02 ± 0.01	[11]
Pellet diameter (mm)	1.57 ± 0.41	0.16 ± 0.05	0.23 ± 0.06	[11]
P/V_av_ (WL^−1^)	0.20	0.44	0.51	[15]
% APA in soluble protein^a^	23 ± 6	15 ± 4	16 ± 5	[11]
APA produced (mgL^−1^)^a^	~ 0.51	~ 0.81	~ 0.81	[11]
Y_total protein/X_, g/g	0.23 ± 0.03	0.34 ± 0.03	0.37 ± 0.04	[11]
C-terminal *O*-mannosylation	2	5	5	[11]

^a^Calculated based on gel densitometry performed on the soluble protein obtained

On the other hand, the OTR is the product of the volumetric oxygen transfer coefficient (k_L_a) and the oxygen concentration difference between the saturated gas–liquid interface and the liquid bulk (C_L_) (OTR = k_L_a[C* − C_L_]). The oxygen gradient between the gas–liquid interface and the liquid bulk results from the oxygen uptake rate (OUR) of the cells; therefore, the OTR can be associated with the microorganism’s respiration activity [29, 30]. We found that a low OTR can activate the metabolic switch. In the present study, we investigated the role of OTR, carbon dioxide transfer rate (CTR), and respiration quotient (RQ) as well as the production of RED, a molecule associated with secondary metabolism, in a recombinant glycoprotein-producing strain of *S. lividans*. We used two irregular shake flask designs and compared the results with a conventional Erlenmeyer flask design.

## Results

### Growth of a recombinant strain of *S. lividans*

**Fig. 1:**
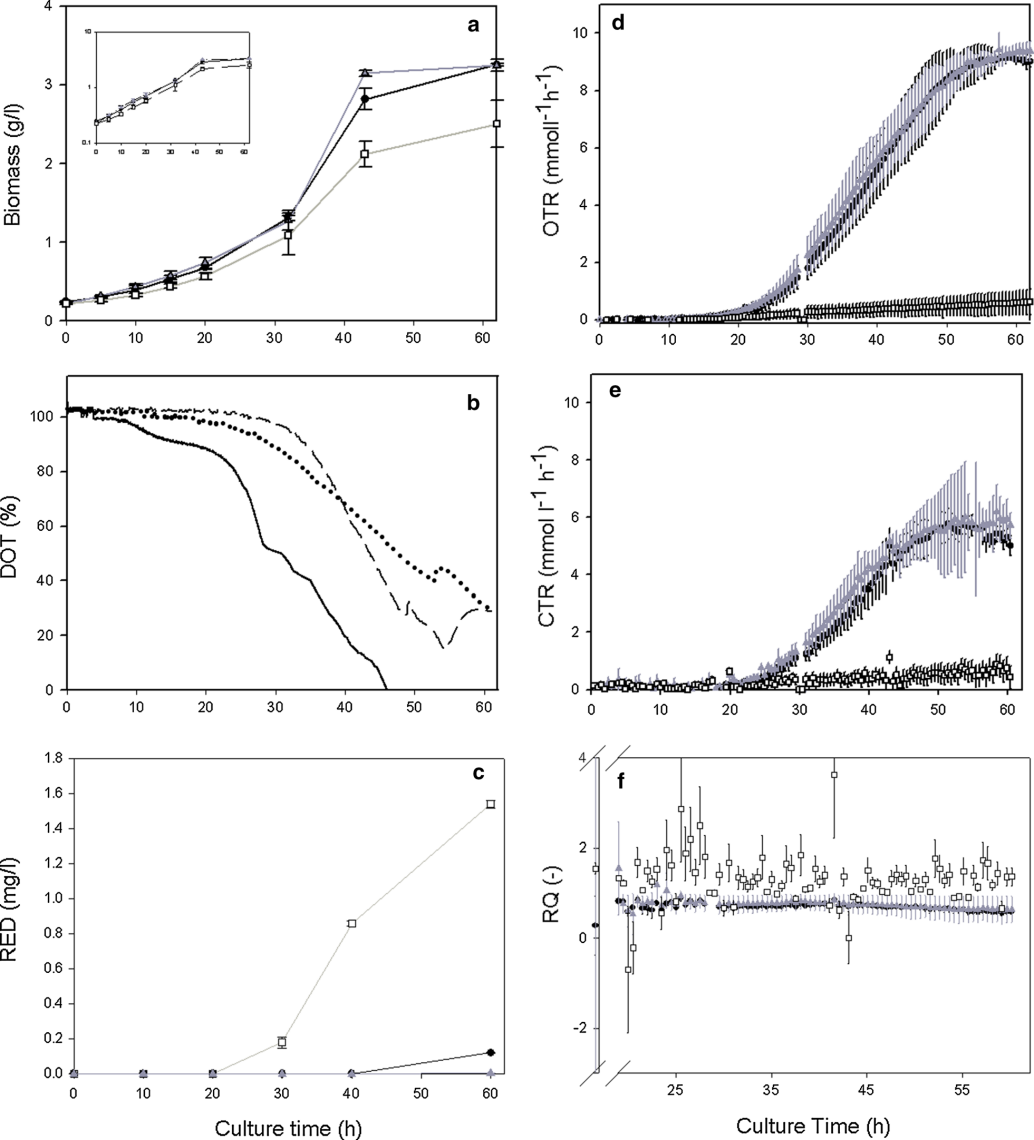
Kinetics of recombinant *S. lividans* producing rAPA from *M. tuberculosis* in conventional normal (NF, squares), coiled (CF, circles), and baffled (BF, triangles) shake flask cultures. **a** Biomass dry weight growth; the inset presents the growth by a logarithmic axis. **b** Characteristic dissolved oxygen tension (DOT) trends in NF (continuous lane), BF (dotted lane), and CF (dashed line). **c** Undecylprodigiosin (RED) production. **d** Oxygen transfer rate (OTR) trends. **e** Carbon transfer rate (CTR) trends. **f** Respiration quotient (RQ). All cultures were carried out at 30 °C, 150 rpm, and 2.5 cm orbital-shaking diameter, in 250-mL shake flasks with 50-mL filling volume. Symbols represent the median and the standard deviation of at least three independents experiments

Shake flasks are widely used in different bioprocesses; however, the impact of flask design has not been extensively studied [31]. Figure 1 shows that the use of different flask designs (NF, CF, and BF) impacts the growth of a recombinant strain of *S. lividans* (biomass), the dissolved oxygen tension (DOT), RED production, and the OTR, CTR, and RQ values. Biomass reached a maximum of 3.2 ± 0.8 g/L in CF and 3.2 ± 0.2 g/L in BF, but only reached 2.5 ± 0.3 g/L in NF (Table 2, Fig. 1a). However, no significant differences in the specific growth rate (μ) were observed among NF, CF, and BF (Table 2). The differences in the final biomass concentrations among NF, CF, and BF, were concurrent with a prior study by our group [15], and with the cultures made with the wild-type strain reaching a maximum biomass concentration of 4.6 ± 0.1 g/L in CF, 4.8 ± 0.1 g/L in BF and in 2.8 ± 0.1 in NF (Table 2, Additional file 1: Figure S1A). Interestingly, the specific growth rates in this study were approximately half of those we previously reported [11, 15], likely due to the age and viability of the master spore bank, which is the same as was used in our earlier work. Prior research has demonstrated that storage conditions may affect viability, spore refractility, heat resistance, desiccation resistance, and the time required for spore germination in complex media [8, 32].

**Table 2:** Stoichiometric and kinetic parameters of *S. lividans* growth, the recombinant glycoprotein production (APA, also known as alanine and proline-rich secreted protein or 45/47 kDa glycoprotein, an antigen from *Mycobacterium tuberculosis*) and RED production in conventional normal, baffled, and coiled flasks (nominal volume of 250 mL and 50 mL filling volume, incubated at 30 °C at a shaking frequency of 150 rpm). A comparison was made with the wild-type strain (WT) (Additional file 1: Figure S1)

Parameter	Shake flask		
	Normal	Coiled	Baffled
Biomass_max_ (g L^−1^)	2.5 ± 0.3	3.2 ± 0.8	3.2 ± 0.2
Biomass_max_ (g L^−1^) WT	2.8 ± 0.1	4.6 ± 0.1	4.8 ± 0.1
µ (h^−1^)	0.055 ± 0.006	0.058 ± 0.005	0.057 ± 0.005
µ (h^−1^) WT	0.069 ± 0.005	0.091 ± 0.004	0.096 ± 0.002
k_L_a (h^−1^)	41.4 ± 5.4	129.9 ± 5.0	87.4 ± 0.6
OTR_max_ (mmol L^−1^ h^−1^)	0.66 ± 0.48	9.16 ± 0.15	9.36 ± 0.28
OTR_max_ (mmol L^−1^ h^−1^) WT	~ 1.6	~ 7.2	~ 9.7
CTR_max_ (mmol L^−1^ h^−1^)	0.77 ± 0.35	5.73 ± 0.77	6.18 ± 0.41
RQ average	1.34 ± 0.49	0.69 ± 0.07	0.75 ± 0.10
UDP (mg L^−1^)	1.540 ± 0.021	0.120 ± 0.002	0.004 ± 0.001
UDP (mg L^−1^) WT	0.61 ± 0.11	0.19 ± 0.05	0.15 ± 0.02
GDP-mannose (ng L^−1^)	1.1 ± 0.1	4.0 ± 1.0	6.0 ± 1.1

The mean and standard deviation for at least three independent experiments are presented

### Oxygen transfer considerations in the growth of a recombinant strain of *S. lividans*

The DOT in shake flask cultures showed a faster decrease in NF than in CF and BF (Fig. 1b). The DOT decreased to 0% after 45 h of culturing in NF, while it remained over 20% in BF and CF, suggesting that there was no oxygen limitation in these setups. In *S. orientalis* and *S. erythraeus* cultures, oxygen limitation in shake flasks acts in an analogous manner to substrate limitation imposed by dissolved nutrients, stimulating secondary metabolite production [33].

An exponentially increasing OTR was observed in CF and BF cultures, reaching similar maximum OTRs of 9.16 ± 0.15 and 9.36 ± 0.28 mmol L^−1^ h^−1^, respectively (Table 2, Fig. 1d). On the other hand, a lower maximum OTR (0.66 ± 0.48 mmol L^−1^ h^−1^), approximately 15 times less than that for CF and BF, was observed in NF. Although biomass growth did not appear to be dramatically affected by OTR (Fig. 1a), the metabolic processes associated with bacterial aggregation, recombinant protein production, and protein *O*-mannosylation appeared to be impacted by the oxygen transfer phenomena as previously reported [11] and corroborated by this work (data not shown).

In order to determine if the metabolic stress associated with the production of the recombinant glycoprotein will impact switching between primary and secondary metabolism, OTR values was evaluated for the wild type strain (Additional file 1: Figure S1). A similar behavior than the recombinant *S. lividans* strain was found, being ~ 7.2 mmol L^−1^ h^−1^ for CF. ~ 9.7 mmol L^−1^ h^−1^ for BF and ~ 1.6 mmol L^−1^ h^−1^ for NF (Table 2, Additional file 1: Figure S1B). Furthermore, as a proof of concept, we measured the OTR in the three shake flask designs using a microorganism with higher respiration activity (*Corynebacterium glutamicum*), and similar OTR trends were obtained (Additional file 1: Figure S2). In BF and CF, the maximum OTR was similar to that of the wild-type strain of *S. lividans* cultures, In together these results suggesting that approximately 10 mmol L^−1^ h^−1^ is the maximum value reachable with these flask designs and culture conditions (150 rpm and 30 °C).

The CTR values in CF and BF cultures followed a similar trend as the OTR but were, on average, 70% lower in terms of empirical value (Table 2, Fig. 1e); however, in NF, the CTR was approximately 40% greater than the OTR, likely due to changes in the microorganism’s metabolism (Table 2, Fig. 1e). The higher value of CTR compared to OTR may be related to respiration activity, which is measured as the respiration quotient (RQ), defined as the ratio of CTR to OTR (Table 2, Fig. 1f). When a culture is undergoing aerobic metabolism, RQ is less than 1.0, as can be seen in CF and BF; however, when changes occur to limit aerobic metabolism, RQ is greater than 1.0, as can be seen in NF [29]. Irregular readings for RQ during the first 20 h (omitted in Fig. 1f) were due to very low cell densities giving non-exact OTR and CTR readings in the RAMOS device.

To further characterize the oxygen transfer in these shake flask designs, the volumetric mass transfer coefficient (k_L_a) was measured (Table 2). As theoretically expected [34, 35], k_L_a values were almost two and three times greater in BF and CF (87.4 ± 0.6 h^−1^ and 129.9 ± 5.0 h^−1^, respectively) compared with NF (41.4 ± 5.4 h^−1^). Then, it can be observed that there are greater oxygen transfers in those alternative designs (BF and CF), which improved the productivity of the recombinant protein and its mannosylation [11].

### RED production and characterization

**Fig. 2:**
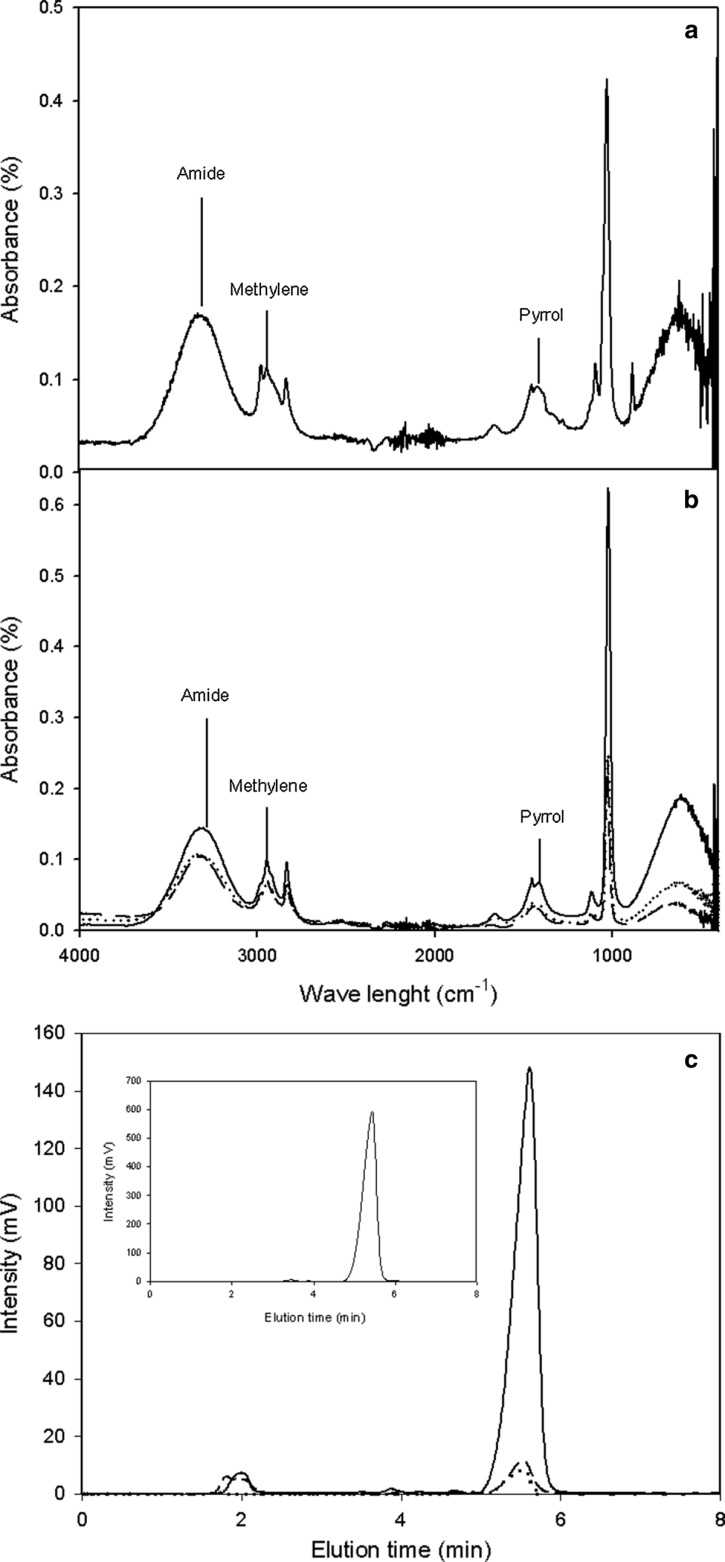
ATR-FTIR structural analysis of undecylprodigiosin. **a** Standard prodigiosin spectrum (C_20_H_25_N_3O_ M.W. 323.44 g/mol, Merck-Sigma-Aldrich, Darmstadt, Germany). **b** ATR-FTIR spectrum from NF, BF, and CF cultures; names above each peak indicate the functional group, CF (dashed line), BF (dotted line), and NF (continuous line). **c** HPLC: Elution profile of undecylprodigiosin, CF in dashed line, BF in dotted line, and NF in continuous line. (Inset: Standard prodigiosin, Merck-Sigma-Aldrich, Darmstadt, Germany)

In NF cultures, an intense red coloration representing RED production was observed by the end of the growth period; this was not observed in CF and BF. RED formation is associated with glucose or phosphate limitation in *Streptomyces* sp. [3, 20], as well as with morphological differentiation [36]. To verify the possible formation of RED in recombinant *S. lividans* cultures, culture samples were qualitatively compared with a commercial prodigiosin standard by ATR-FTIR (Fig. 2a), as well as quantitatively and kinetically analyzed by HPLC in cultures carried out in the three shake flask designs (Figs. 1c, 2b).

The ATR-FTIR spectrum showed the characteristic peaks for RED at 1565 cm^−1^ corresponding to a pyrrole group, 2909 cm^−1^ corresponding to a methylene group, and 3463 cm^−1^ corresponding to the amide group (Fig. 2a), consistent with those previously reported [33]. Although ATR-FTIR is not a quantitative technique, the peak intensity for the characteristic groups of RED was at least two-fold higher for NF than for CF and BF, suggesting an increase in the production of the red pigment towards the end of the culture. Additionally, a quantitative approach was carried out through HPLC using a commercial prodigiosin as a standard (Fig. 2b, inset). RED was detected in NF after 30 h of culturing (Fig. 1c), while in CF and BF the production was observed only after 60 h of culturing. At the end of the culture period, 1.540 ± 0.021 mg/L of RED was measured in NF, while 0.004 ± 0.001 and 0.120 ± 0.002 mg/L were measured in BF and CF, respectively (Table 2). Interestingly, the design of flask with low oxygen transfer (NF) increases the productivity of RED, inversely to the productivity of the recombinant protein and its mannosylation that occurs in the designs of higher oxygen transfer (BF and CF). Moreover RED production was evaluated at the end of wild-type strain cultures, being 0.19 ± 0.05 mg/L for CF, 0.15 ± 0.02 mg/L for BF and 0.61 ± 0.11 mg/L for NF (Table 1).

## Discussion

A complex series of molecular alterations is associated with the metabolic switch in *Streptomyces* [3]. For example, in *S. coelicolor*, the nitrogen metabolism gene cluster is downregulated, while the regulatory genes *phoP*, *phoU*, and *phoR* in the PHO regulon are upregulated after the metabolic switch [3, 6]. Moreover, the biosynthetic gene cluster for RED and actinorhodin are upregulated, while the up- or downregulation of gene clusters responding to nutrient depletion depleting-nutrient gene clusters does not occur at the same time as antibiotic biosynthesis, with the latter being upregulated during the late stationary phase of growth [3]. Another example is the modulatory effect that PhoU (phosphate-specific transport system accessory protein) has on ACT and RED biosynthesis through phosphate regulation [37].

*Streptomyces lividans* is the bacterial system of choice within the genus *Streptomyces* for heterologous protein and glycoprotein production, even though the most studied species of the genus is *S. coelicolor* [14]. This is mainly due to *S. lividans* exhibiting less extracellular proteolytic activity, as well as the lack of a strong restriction system in *S. coelicolor* [7, 12, 38]. Previously, we showed that flask design does not significantly affect biomass growth kinetics [11], as also seen in this work; however, it does significantly affect recombinant glycoprotein production and protein *O*-mannosylation, also observed here for the NF, CF, and BF flasks. Here, we showed that the OTR and CTR were dramatically different in NF compared with CF and BF. However, the specific growth rate was unchanged among the flask designs. Normally, in the late stationary phase, there are very few cells with high respiratory activity levels [39]; this is a likely cause for the reduction of OTR and the overproduction of RED observed in the NF design. The OTR and CTR measured in NF, the only culture that reached 0% DOT, were approximately 15 times lower than in CF and BF; as a consequence, up to 12 and 385 times more RED was measured in NF than in BF and CF, respectively. No such dramatic differences in RED were seen with the wild-type strain (Table 1). This indicates that the metabolic burden associated with the production of the recombinant glycoprotein, in addition to the availability of oxygen is playing an important role in the production of RED and in the metabolic switch [40].

When baffles or coiled springs are added to flasks, an entrapment phenomenon arises such that small drops are formed when the liquid collides with the baffles or the springs. In addition, small air bubbles are formed and entrained into the liquid. These drops and bubbles represent an important aspect of oxygen transfer that does not occur in NF [41]. Even when BF and CF appeared similar in biomass growth, OTR, CTR, RQ, production, and *O*-mannosylation of the recombinant protein, the volumetric mass transfer coefficient (k_L_a) reached in CF was higher than that obtained in BF (Table 2), while the power input was higher in BF than in CF [15]. This suggests that global mass and momentum operational parameters (k_L_a, P/V, and rpm, among others) cannot solely explain the behavior of a fluid in a singular design of a shaken bioreactor, but is rather largely a function of the dissimilar flow pattern of the liquid bulk as was reported previously [41, 42]. The OTR and k_L_a have been widely used in scaling up biotechnological processes in bioreactors. However, there have only been a few reports on the OTR, DOT, and k_L_a in shake flasks [4]; moreover, to our knowledge, none have reported the OTR or k_L_a in an irregular design, such as with the homemade BF and CF in this study.

To explain the increased CTR (compared to OTR) in NF, the fermentative pathway was considered. In *S. coelicolor*, the presence of the lactate dehydrogenase gene (SCO2118) [43] has been reported. In *S*. *griseus*, lactate production has been reported, supporting the idea of a lactic acid fermentation mechanism [44]. Furthermore, it is known that *Streptomyces* sp. contain nitrate reductase genes in order to use nitrogen as the terminal electron acceptor, but there is no reported evidence of its usage [45]. To test the hypothesis that lactic acid fermentation was occurring in NF cultures, lactate was measured during cultures, but no lactate was detected.

**Fig. 3:**
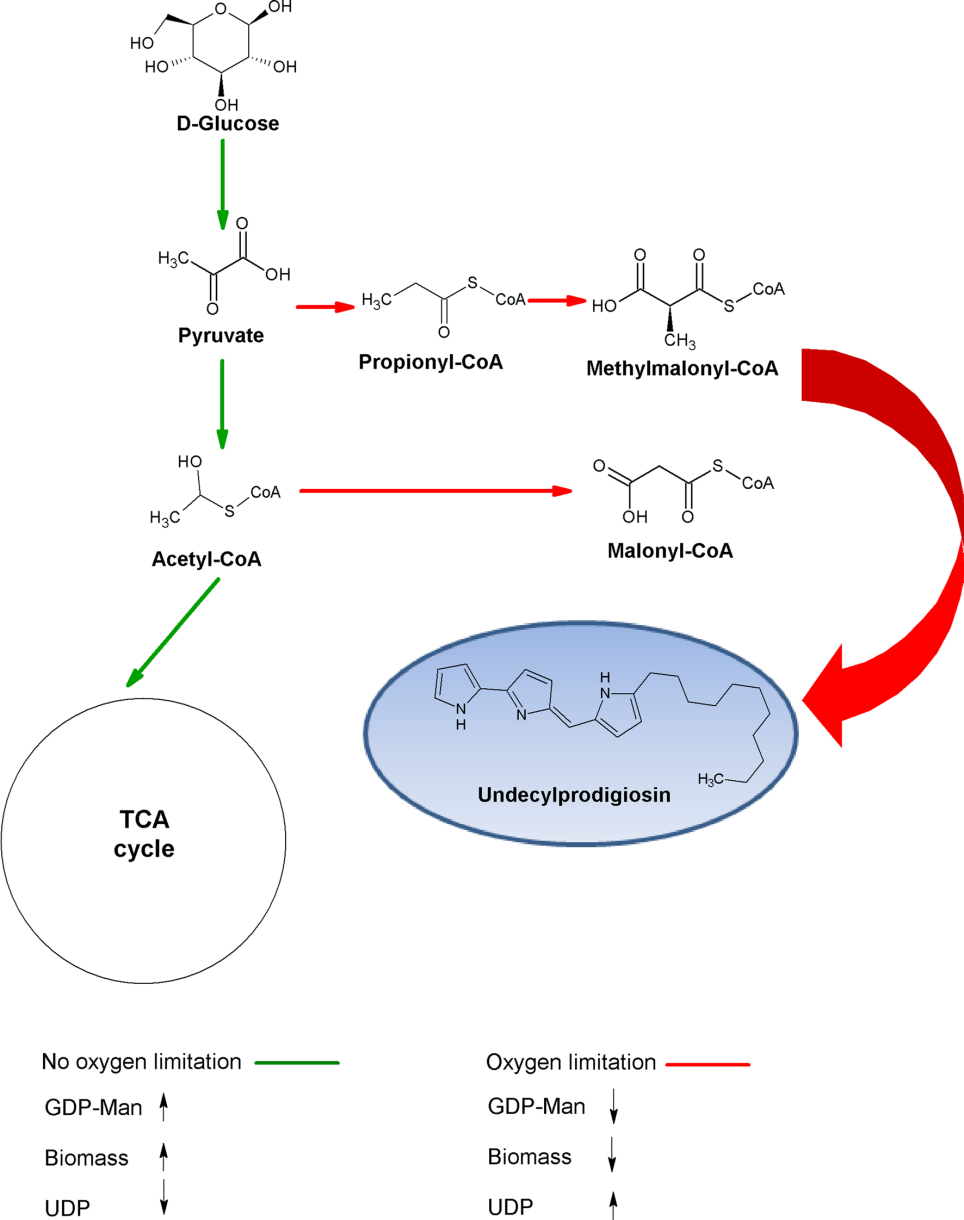
Metabolic pathway proposed when no oxygen limitation (solid line) or oxygen limitation (dotted line) occurs in recombinant *S. lividans* cultures

*Streptomyces* is a well-known producer of antibiotics including actinorhodin, RED, and calcium-dependent antibiotics [43, 46]. The synthesis of these compounds produces carbon dioxide resulting from amino acid and fatty acid catabolism [47]. The conversion of 1 mol of malonyl CoA to RED produces ~ 8 mol of carbon dioxide [48]. Furthermore, the synthesis of RED requires l-proline, most of which is produced de novo, requiring ~ 3 molecules of NADH per one molecule of l-proline, and ~ 30 mmol of NADH is consumed per gram of RED [49]. As suggested by the RQ data, the TCA cycle is performed at a higher rate in CF and BF than in NF. Butler et al. [50] proposed that a high carbon flux through the TCA cycle affects the availability of carbon for antibiotic synthesis. In RED synthesis, NADH is used, decreasing the reducing power allotted for ATP and GTP production. GTP is involved in the *O*-mannosylation pathway [51]; hence, its scarcity in NF cultures may explain the decrease in *O*-mannosylation previously observed [11]. In this study, 6.0 ± 1.1 and 4.0 ± 1.0 ng/L of GDP-mannose was observed in BF and CF, respectively, while only 1.1 ± 0.1 ng/L was measured in NF (Table 2); these results indicate an increase in metabolic flux through the central carbon metabolic pathway [52]. A schematic of the pathway is shown in Fig. 3. When sufficient oxygen is available in culture media, the carbon source is mainly used for biomass and recombinant glycoprotein formation; on the other hand, when oxygen is limited, secondary metabolite production is enhanced.

## Conclusions

NFs are widely used in research, but the inclusion of baffles or stainless steel springs in *Streptomyces* sp. cultures is often done to avoid pellet formation and to increase the oxygen transfer that could lead to metabolic improvements and enhanced recombinant protein production. To our knowledge, no correlation has been proposed between oxygen or carbon transfer, recombinant glycoproteins and secondary metabolite production in *Streptomyces* sp. cultures. In this work, we report significant differences in the OTR, CTR, and RQ in NF, CF, and BF. The suboptimal aeration conditions in NF resulted in the overproduction of RED and a decrease in glycoprotein yield and *O*-mannosylation in a recombinant strain of *S. lividans.*

The higher OTR reached in CF and BF allowed *S. lividans* to avoid some of the metabolic switch that occurred in NF and that has been reported in other cultures of *Streptomyces* sp. [2], observed in both, recombinant and wild type strains. We observed an RQ value greater than 1 in NF cultures due to an increase in carbon dioxide liberation, probably due to the biosynthesis of secondary metabolites such as RED. In line with this, our results indicated that under lower OTR (~ 2 mmol L^−1^ h^−1^) conditions, the biosynthesis of RED was up to 12 and 385 times greater in NF compared with BF and CF. RED production started when oxygen was limited in NF; in contrast, there was no significant oxygen limitation in BF and CF, so RED production was detected at a much later time point and at lower concentrations. In addition to the previously published data regarding the metabolic switch triggered by phosphate [2, 5, 18, 19] or glucose depletion [53], we provide evidence in this study that oxygen limitation can also influence the metabolic switch from primary to secondary metabolism in *Streptomyces* sp.

The increase in RED synthesis and the high carbon dioxide production caused by low oxygen availability could also decrease recombinant glycoprotein synthesis by lowering the productivity of extracellular proteins, as previously reported [11]. In addition, during RED formation in NF, a higher number of precursors such as GTP may be used, limiting *O*-mannosylation of recombinant proteins [11].

Together with previous reports [11–15], this study contributes data on useful culture parameters, such as OTR, CTR, RQ, and k_L_a, as well as an increased understanding of recombinant glycoprotein and secondary metabolite production in the three shake flask designs used for *S. lividans* cultivation.

## Materials and methods

### Microorganism, culture conditions, and analytical methods

Wild type *S. lividans* 66 strain 1326 was transformed with plasmid pIJ6021MT-45 carrying the *apa* gene under a thiostrepton-inducible promoter and a kanamycin resistance gene [10, 11]. Spores were germinated in YT medium for 6 h and inoculated to obtain 0.025 A.U. (600 nm, DU730, Beckman Brea, CA, USA). The cultures were carried out in triplicates in three different Erlenmeyer shake flask designs: normal flask (NF); coiled flasks (CF), which are normal Erlenmeyer flasks with a 30-cm stainless-steel spring (1.3 cm diameter, 19 sw gauge) inserted; and homemade baffled flasks (BF) with three baffles 4 cm tall and 2 cm wide, identical to those used by Gamboa-Suasnavart et al. [11]. All cultures were carried out at 30 °C with shaking at 150 rpm with an orbital diameter of 2.5 cm (C25 Incubator Shaker, New Brunswick Scientific, Edison, NJ, USA) in 250-mL flasks containing 50 mL of Luria–Bertani (LB: 10 g/L tryptone, 5 g/L yeast extract, and 5 g/L NaCl) broth, modified with 34% w/v sucrose with kanamycin (50 µg/mL). The inducer (10 µg/mL thiostrepton) was added midway during the exponential phase. Biomass was evaluated by dry weight; 10 mL of culture was filtered through a 0.45-μm pore size membrane (Merck-Millipore, Billerica, MA, USA), which was washed once with one volume of distilled water. The mycelium obtained was dried for 24 h in an oven at 55 °C, placed for 2 h in a desiccator, and then weighed [11]. For wild-type cultures *S. lividans* 66 strain 1326 were used with the same culture conditions.

### Online measurement of the oxygen transfer rate (OTR) and carbon transfer rate (CTR)

Three BF, three CF, and two NF flasks with a specially designed neck were used for each experiment with the recombinant strain of *S. lividans* in the RAMOS device [29, 31]. Aeration cycles of 10 min measuring and 20 min rinsing phases were employed to mimic the gas phase concentrations in NF, BF, and CF with cotton plugs [29, 54]. The OTR, as the result of oxygen uptake, and the CTR, as the result of CO_2_ production, were measured by partial pressure sensors in the headspace during the cultivation [29, 54].

In the wid-type strain cultures, the OTR was achieve by means of BlueSense BCP-O2 probe (BlueSens, Germany) with BlueVis software (Version 1.0.0.1) for oxygen concentration acquisition. The probe was located in the shake flask headspace for monitoring gas changes. The shake flasks with GL 45 threated ports for probe installation were used in all the cultures. Gas concentration data was used for OTR calculation according equations sets by vendor with some corrections.

### Online measurement of DOT

Measurement of the DOT in the recombinant strain of *S. lividans* cultures was carried out and recorded online with the oxygen optical meter Fibox 3 using a PSt3 sensor (PreSens, Regensburg, Germany). The sensor was glued to the bottom of each Erlenmeyer flask [55]. The optical sensor was calibrated to 0% air saturation by using culture media containing Na_2_SO_3_ (0.3 M) and CoCl_2_ (≤ 5 × 10^−7^ M), and to 100% by using culture media in equilibrium with the ventilation air flow. Flasks were shaken during calibration using the same incubator that was used for cultivation (C25, New Brunswick Scientific, Edison NJ, USA) and filled with the same volume (50 mL), ensuring that the optical sensor was always covered with liquid.

### Volumetric mass transfer coefficient (k_L_a) measurement in shake flasks

Measurements of k_L_a were performed at the same culture conditions (150 rpm, 30 °C, 250 mL shake flasks filled with 50 mL of distilled water). The dissolved oxygen was reduced to zero by using Na_2_SO_3_ (0.3 M) and CoCl_2_ (≤ 5 × 10^−7^ M) [55]. Agitation was started when there was no more oxygen in the water, and then DOT was recorded online. The mass transfer coefficient (k_L_a) was obtained as linear slope resulting from plotting the logarithmic expression against time, as shown in Eq. 1. Only data measured between 10% and 60% DOT were used for k_L_a estimation [32].

1 $$\ln \left( {\frac{{C_{L}^{*} - C_{L2} }}{{C_{L}^{*} - C_{L1} }}} \right) = - k_{L} a \times \left( {t_{2} - t_{1} } \right)$$

### Attenuated total reflection Fourier transform infrared spectroscopy (ATR-FTIR) of RED

A Shimadzu IRAffinity-1S FTIR spectrometer (Shimadzu, Kyoto, Japan) with a Specac Quest ATR diamond accessory (Specac Limited, England) was used to obtain the ATR-FTIR spectra of the hydrated thin-film of undecylprodigiosin (extracted from biomass using methanol acidified with 3 N HCl to pH 2.0) in a wave number range of 4000 cm^−1^ to 500 cm^−1^. A total of 40 interferograms were collected and averaged. Prodigiosin (C_20_H_25_N_3O_ M.W. 323.44 g/mol, Merck-Sigma-Aldrich, Darmstadt, Germany) was used as a standard. ATR-FTIR analysis was done in triplicate for three independent cultures for each shake flask culture condition [56].

### RED quantification by high-performance liquid chromatography (HPLC)

RED was extracted from biomass using methanol acidified with 3 N HCl to pH 2.0. A standard curve was generated with prodigiosin (C_20_H_25_N_3O_ M.W. 323.44 g/mol, Merck-Sigma-Aldrich, Darmstadt, Germany) at concentrations of 0.25, 0.0125, 0.006, and 0.001 mg/mL. Samples were filtered through a 0.45-μm pore size membrane (Merck-Millipore, Billerica, MA, USA), and then quantified by HPLC (Shimadzu, Kyoto, Japan) with a UV detector (535 nm). Aliquots were analyzed by isocratic elution in a solution of 10% 0.5 M PBS, 75% methanol, and 15% water on a C-8 Eclipse XDB column (5 µm, 4.6 × 150 mm; Agilent, USA) with a flow rate of 0.8 mL/min at 30 °C [57].

### GDP-mannose detection by high-performance anion-exchange chromatography

A 5-mL sample of biomass was centrifuged at 15,000×*g* (Eppendorf Mod. 5804-R, Hamburg, Germany) and then washed twice with PBS. The biomass was frozen and stored at -80 °C until subsequent sonication. Extraction was performed with a solution composed of 50% ethanol and 10 mM ammonium phosphate, pH 3.0. GDP-mannose detection was conducted by high-performance anion-exchange chromatography (Shimadzu, Kyoto, Japan) using a detector at 254 nm and a Varian Polaris C 18-A (4.6 × 150 mm) column. Monopotassium phosphate (KH_2_PO_4_) buffer (0.5 M, 50%) with H_2_O was the mobile phase, the flow rate was set at 1 mL/min, and the procedure was carried out at 50 °C [52].

### Statistical analysis

All cultures were carried out at least in triplicate. Independent samples and multiple-comparison tests were used to estimate statistical significance of differences in the culture parameters (two-way analysis of variance [ANOVA] and Tukey’s Post Hoc test were used). A threshold significance level of 0.05 was applied.

## Additional file

Additional file 1: Figure S1. Kinetics of *S. lividans* wild type in conventional normal (NF, squares), coiled (CF, circles), and baffled (BF, triangles) shake flask cultures. A: Biomass dry weight growth; the inset presents the growth by a logarithmic scale. B: Oxygen transfer rate (OTR) trends. **Figure S2.** OTR measurements for C. glutamicum cultures in CF (circles), BF (triangles), and NF (squares). Cultures were carried out at 30°C, 150 rpm in 250-mL shake flasks with 50 mL filling volume.

## Abbreviations

C_L_: dissolved oxygen concentration (mmol L^−1^)
C*: dissolved saturated oxygen concentration (mmol L^−1^)
DOT: dissolved oxygen tension (%)
k_L_a: volumetric mass transfer coefficient (h^−1^)
NF: normal Erlenmeyer flask
CF: coiled Erlenmeyer flask
BF: baffled Erlenmeyer flask
P/V: power input (W L^−1^)
RED: undecylprodigiosin
OTR: oxygen transfer rate (mmol L^−1^ h^−1^)
OUR: oxygen uptake rate (mmol L^−1^ h^−1^)
CTR: carbon dioxide transfer rate (mmol L^−1^ h^−1^)
RQ: respiration quotient (CTR/OTR)
