# Supplementary material for: The metabolic switch can be activated in a recombinant strain of Streptomyces lividans by a low oxygen transfer rate in shake flasks
Source: Microb Cell Fact. 2018 Nov 28;17:189. doi: 10.1186/s12934-018-1035-3 (PMC6260694; doi:10.1186/s12934-018-1035-3)
Supplement: Supplementary file 1 — Additional file 1: Figure S1. Kinetics of S. lividans wild type in conventional normal (NF, squares), coiled (CF, circles), and baffled (BF, triangles) shake flask cultures. A: Biomass dry weight growth; the inset presents the growth by a logarithmic scale. B: Oxygen transfer rate (OTR) trends. Figure S2. OTR measurements for C. glutamicum cultures in CF (circles), BF (triangles), and NF (squares). Cultures were carried out at 30°C, 150 rpm in 250-mL shake flasks with 50 mL filling volume. [file 12934_2018_1035_MOESM1_ESM.pdf]

The metabolic switch can be activated in a recombinant strain of *Streptomyces lividans* by a low oxygen transfer rate in shake flasks

Ramsés A. Gamboa-Suasnavart[1], Norma A. Valdez-Cruz[1], Gerardo Gaytan-Ortega[1], Greta I. Reynoso-Cereceda[1], Daniel Cabrera-Santos[1], Lorena López-Griego[1], Wolf Klöckner[2,3], Jochen Büchs[2] and Mauricio A. Trujillo-Roldán[1*]

1. Programa de Investigación de Producción de Biomoléculas, Unidad de Bioprocesos, Departamento de Biología Molecular y Biotecnología, Instituto de Investigaciones Biomédicas, Universidad Nacional Autónoma de México. AP. 70228, Ciudad de México, CP. 04510, México.

2. Department of Biochemical Engineering (AVT.BioVT), RWTH Aachen University of Technology, Forckenbeckstraße 51, D-52074 Aachen, Germany.

3. Bayer AG, Engineering and Technology, Chempark, 51368 Leverkusen, Germany.

*** Corresponding author: Dr. Mauricio A Trujillo-Roldán**

Unidad de Bioprocesos, Instituto de Investigaciones Biomédicas, Universidad Nacional Autónoma de México, AP. 70228, México D.F., CP. 04510, México, Tel.: +525556229192; maurotru@gmail.com, maurotru@biomedicas.unam.mx

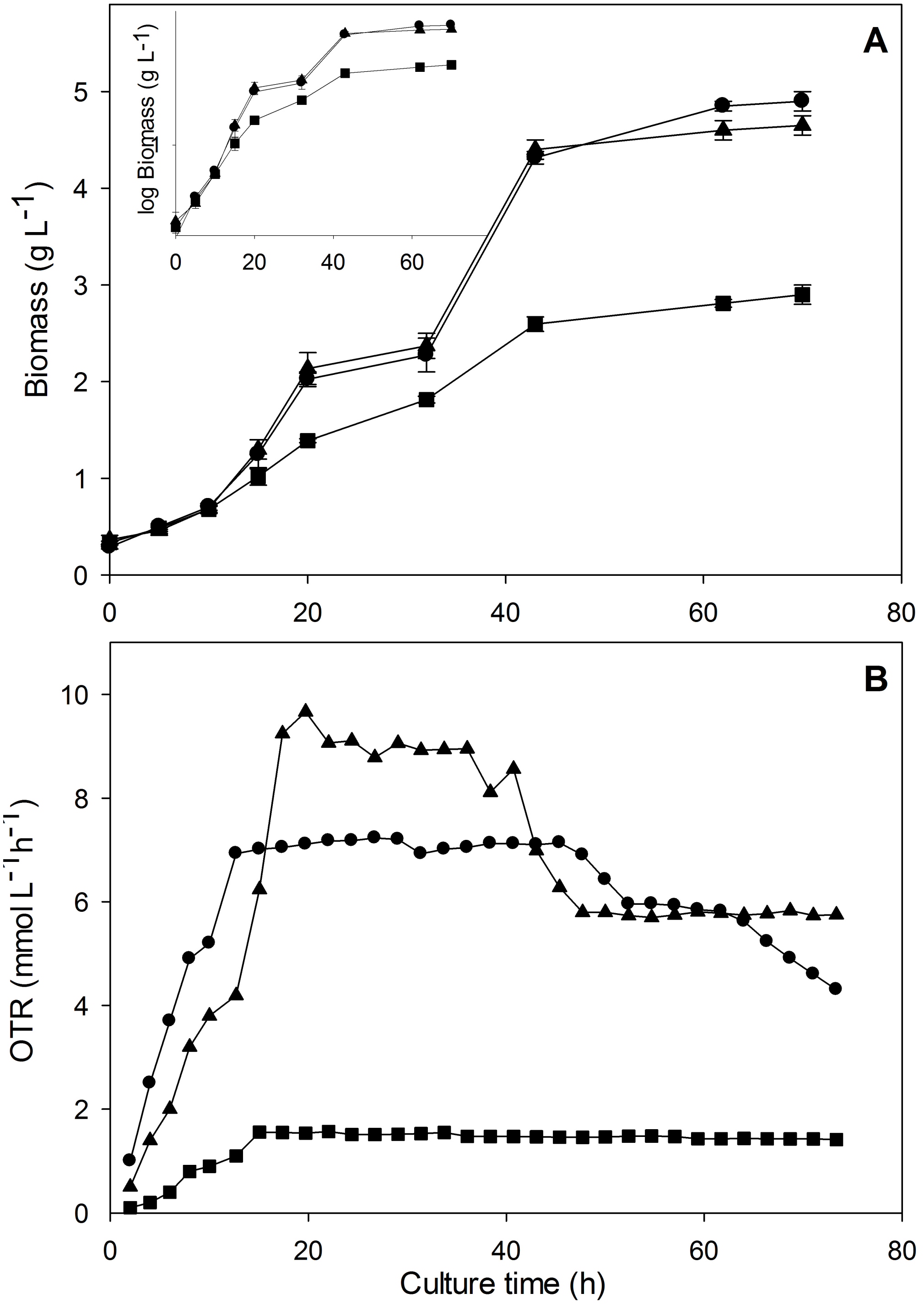

Figure S1. Kinetics of *S. lividans* wild type in conventional normal (NF, squares), coiled (CF, circles), and baffled (BF, triangles) shake flask cultures. **A:** Biomass dry weight growth; the inset presents the growth by a logarithmic scale. **B:** Oxygen transfer rate (OTR) trends.

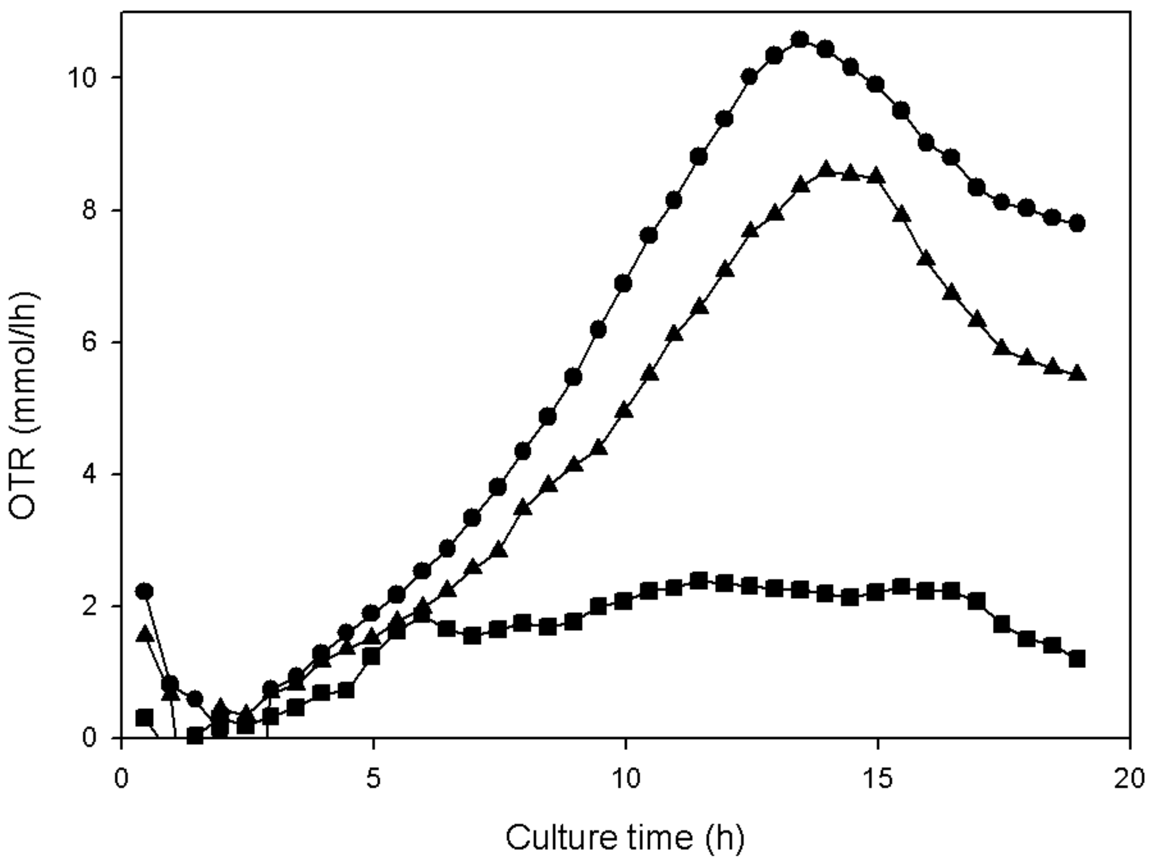

Figure S2. OTR measurements for *C. glutamicum* cultures in CF (circles), BF (triangles), and NF (squares). Cultures were carried out at 30°C, 150 rpm in 250-mL shake flasks with 50 mL filling volume.
